# The effects of herding and dispersal behaviour on the evolution of cooperation on complete networks

**DOI:** 10.1007/s00285-024-02148-y

**Published:** 2024-10-06

**Authors:** Hasan Haq, Pedro H. T. Schimit, Mark Broom

**Affiliations:** 1 Department of Mathematics, City, University of London, 10 Northampton Square, London, EC1V 0HB UK; 2 Informatics and Knowledge Management Graduate Program, Universidade Nove de Julho, Rua Vergueiro, 235/249, São Paulo, SP CEP: 01504-000 Brazil

**Keywords:** Evolutionary game theory, Evolutionary graph theory, Structured populations, Multiplayer games, Herding, Dispersal, 91A43, 91A22

## Abstract

Evolutionary graph theory has considerably advanced the process of modelling the evolution of structured populations, which models the interactions between individuals as pairwise contests. In recent years, these classical evolution models have been extended to incorporate more realistic features, e.g. multiplayer games. A recent series of papers have developed a new evolutionary framework including structure, multiplayer interactions, evolutionary dynamics, and movement. However, so far, the developed models have mainly considered independent movement without coordinated behaviour. Although the theory underlying the framework has been developed and explored in various directions, several movement mechanisms have been produced which characterise coordinated movement, for example, herding. By embedding these newly constructed movement distributions, within the evolutionary setting of the framework, we demonstrate that certain levels of aggregation and dispersal benefit specific types of individuals. Moreover, by extending existing parameters within the framework, we are not only able to develop a general process of embedding any of the considered movement distributions into the evolutionary setting on complete graphs but also analytically produce the probability of fixation of a mutant on a complete N-sized network, for the multiplayer Public Goods and Hawk–Dove games. Also, by applying weak selection methods, we extended existing previous analyses on the pairwise Hawk–Dove Game to encompass the multiplayer version considered in this paper. By producing neutrality and equilibrium conditions, we show that hawks generally do worse in our models due to the multiplayer nature of the interactions.

## Introduction

Evolutionary game theory has demonstrated its versatility as a multifaceted mathematical modelling tool for understanding the evolution of, and behaviour of biological populations. The classical evolutionary models focused on infinite, well-mixed populations, assuming equal probabilities of interaction among individuals, often involving pairwise contests (Maynard Smith 1982). In certain scenarios, pairwise interactions were absent, and instead, games involving the entire population, known as "against the field" games, were modelled. The original models of evolutionary game theory introduced the concept of an individual’s fitness being dependent on the frequency of their types within the population. These models employed either a static analysis of Evolutionarily Stable Strategies (ESS) (Maynard Smith and Price 1973), the biological extension of the Nash Equilibrium from classical game theory (Nash 1951) or a dynamic analysis involving the replicator equation (Hofbauer et al. 1979; Hofbauer and Sigmund 1998; Taylor and Jonker 1978) which examined how the population composition changes over time.

While simple models such as the sex ratio game have been utilised to explain biological phenomena (Darwin 1874; Fisher 1930; Hamilton 1967), they rely on the unrealistic assumption of infinite populations as real populations are finite in size. Consequently, the Moran process (Moran 1958, 1962) was adapted to incorporate frequency-dependent fitness, leading to the study of evolutionary processes in finite populations. More recently, the approach of modelling the evolution of finite populations through a graph, where individuals exclusively interact with their neighbours, was introduced as evolutionary graph theory (Lieberman et al. 2005). In this framework, individuals are situated on the vertices of a graphical structure and engage in pairwise contests with their connected neighbours. These interactions determine the individuals’ fitness and govern the population’s dynamics during the evolutionary process. The significant advantage of evolutionary graph theory lies in its ability to consider a wide range of population structures (Antal and Scheuring 2006; Broom and Rychtar 2008; Maciejewski 2014; Hindersin and Traulsen 2014; Cuesta et al. 2017). Both population structure and evolutionary dynamics play influential roles in population evolution (Santos and Pacheco 2006; Broom and Rychtar 2008; Voorhees 2013; Tkadlec et al. 2020) in fact, heterogeneous structures are pivotal in facilitating the formation of clusters of cooperators (Li et al. 2013).

However, a limitation of evolutionary graph theory is its pairwise modelling of interactions rather than considering a more realistic arbitrary multiplayer game scenario, thus lacking adaptability and realism. To address this limitation, recent research papers have developed a comprehensive modelling approach that allows for the study of structured population evolution involving multiplayer contests which we denote as the Broom–Rychtá$\hat{\textrm{r}}$ framework (Broom and Rychtar 2012; Broom et al. 2015, 2019). Evolutionary multiplayer games were first introduced by Palm (1984) and further developed by, by Broom et al. (1997), see also Bukowski and Miekisz (2004) and Gokhale and Traulsen (2014). These contests between individuals employ standard games such as Public Goods, Hawk–Dove, and Prisoner’s Dilemma (Ohtsuki et al. 2006; Santos et al. 2008; van Veelen and Nowak 2012; Hadjichrysanthou et al. 2011; Broom and Rychtar 2013). A typical application of the framework is in Broom et al. (2015) which utilises the territorial raider model (an extension of the Broom–Rychtá$\hat{\textrm{r}}$ framework) as the underlying structure of the population and models the interactions between individuals using three games: Public Goods, Hawk–Dove, and Fixed Fitness under a standard BDB dynamics (invasion process) where an individual is selected to reproduce first with probability proportional to their fitness relative to the total population. Following this, the death event occurs where the offspring randomly replaces another member of the parent’s group (see Sect. 2.3). Within this model, individuals move independently i.e. without any influence from past movements (history-independent) nor from other individuals’ movements (row-independent). This type of movement has been frequently considered (Broom et al. 2015; Pattni et al. 2017; Schimit et al. 2019, 2022) alongside history-dependent movement models (Pattni et al. 2018; Erovenko et al. 2019). What has yet to be considered is row-dependent movement, where individuals move in a manner in which they take into account the current movement decisions of other individuals within the population. Broom et al. (2020) developed various mechanisms that represent coordinated movement. These realistic movement mechanisms characterise behaviours displayed by animals (Schmidt et al. 1997; Buhl et al. 2006; Marker et al. 2008) which was explained in detail in Broom et al. (2020).

The purpose of this paper is to develop a methodology to enable us to embed these newly developed row-dependent movement models from Broom et al. (2020) into the evolutionary setting of Broom et al. (2015) on complete graph structures. By doing this, we explore the consequences coordinated movement has on the evolution of cooperation.

## The Broom–Rychtá$\hat{\textrm{r}}$ framework

The modelling framework originated in Broom and Rychtar (2012) and is a very general and versatile methodology. However, we omit the intricate details which can be found in Broom and Rychtar (2012). The framework contains three core components: the population structure, the evolutionary dynamics and the multiplayer games. We first explain the fully independent model of this framework in which individuals move independently of each other and of any past movements and a particular case from the framework, the territorial raider model introduced in Broom and Rychtar (2012) and further developed (Broom et al. 2015; Pattni et al. 2017).

### The fully independent model

**Fig. 1:**
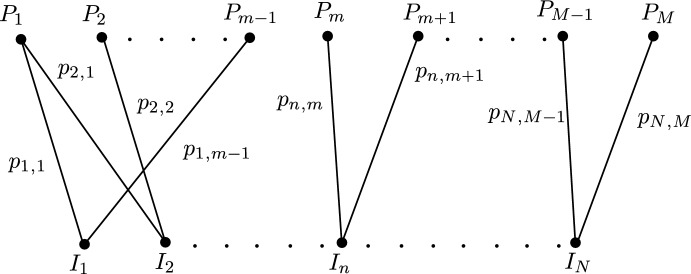
The fully independent model from Broom and Rychtar (2012). There are *N* individuals who are distributed over *M* places such that $I_n$ visits place $P_m$ with probability $p_{nm}$. Individuals interact with one another when they meet, for example, $I_1$ and $I_2$ can interact with one another when they meet in $P_1$

We first describe the fully independent model. Consider a population of *N* individuals $I_1,...,I_N$ who can move around *M* places $P_1,...,P_M$. The probability of individual $I_n$ being at place $P_m$ is denoted by $p_{nm}$; see Fig. 1 for a visual representation using a bi-partite graph. Individuals move along the graph according to their own movement distributions and form groups on the vertices of the graph. Let *G* denote a group of individuals, then $\chi (m,G)$, the probability of group *G* forming at place $P_m$ is given by

1 $$\begin{aligned} \chi (m,G) = \prod _{i \in G}p_{im}\prod _{j \notin G}(1 - p_{jm}). \end{aligned}$$

When a group of individuals is formed, they interact with one another via a multiplayer game. Individual $I_{n}$ receives a payoff that depends upon the composition of the group *G* itself and the place $P_{m}$ the group is present in, denoted by $R_{n,m,G}$. Individual $I_{n}$’s average fitness is calculated by considering all payoffs they can receive averaged over all possible groups and places,

2 $$\begin{aligned} F_{n} = \sum \limits _{m}\sum \limits _{\begin{array}{c} G \\ n \in G \end{array}}\chi (m,G)R_{n,m,G}. \end{aligned}$$

### The territorial raider model

**Fig. 2:**
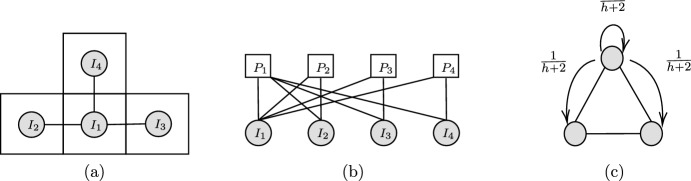
The territorial raider model of Broom and Rychtar (2012), Broom et al. (2015). **a** Population structure is represented using a graph where vertices represent individuals and places. Individual $I_n$ lives in place $P_n$ and can visit any neighbouring places. For example, the home place of $I_1$ is $P_1$ but can visit places $P_2$,$P_3$, and $P_4$. **b** An alternative visualization on a bi-partite graph where individuals and places are separated. **c** An example of the territorial raider model for a well-mixed population of three individuals. The probability any individual stays at home is $\frac{h}{h+2}$ and the probability they move to a neighbouring place is $\frac{1}{h+2}$

Different examples using the fully independent model were developed in Broom and Rychtar (2012). The most significant of these is the territorial raider model, see Fig. 2, which has been extensively explored (Broom et al. 2015; Pattni et al. 2017; Schimit et al. 2019). This model acts as a basis for the work in this paper. In the territorial raider model, there are *N* individuals who can move and interact with other individuals at *M* places. It is assumed individual $I_{i}$ lives at place $P_{m}$ throughout the entire evolutionary process. In the original territorial raider model from Broom and Rychtar (2012) there was a one-to-one correspondence between individuals and places, although this was generalised in Pattni et al. (2017). The amount of time an individual spends on their home vertex is governed by a global home fidelity parameter *h*, which measures the preference individuals have towards staying on their home vertex. The higher *h* is, the more likely individuals are to stay at home and, therefore, less likely to move and interact with other individuals and vice-versa. Given an individual $I_{i}$ with *d* neighbouring places, the probability of $I_{i}$ staying home is $h/(h+d)$ and moving is $d/(h+d)$. If $h = 1$, this represents an indifference individuals have between all reachable places and, therefore, equally likely to visit any of them.

### Evolutionary dynamics

An evolutionary graph (Lieberman et al. 2005; Nowak 2006; Pattni et al. 2015; Möller et al. 2019) is a graph with an associated weighted adjacency matrix $\textbf{W} = (w_{ij})$ where $w_{ij} \in [0, 1)$ is referred to as the replacement weight which governs which members of the population can replace each other. Every vertex $v_{n}$ of the evolutionary graph is occupied by exactly one individual and if $w_{ij} > 0$, then the individual on $v_{i}$ can replace the current individual on $v_{j}$ by placing a copy of itself onto the vertex. The weights are often selected to ensure that the evolutionary graph is strongly connected i.e. there is a finite path between vertices $v_i$ and $v_j$.

A general set of evolutionary dynamics for the Broom–Rychtá$\hat{\textrm{r}}$ framework, analogous to the corresponding evolutionary graph theory dynamics, was developed in Pattni et al. (2017). We note that this process is an idealisation of the original evolutionary process described in Broom et al. (2015), which is represented by the simulations in Sect. 5, allowing for analytical results to be considered. It was identified in Pires et al. (2023) that under certain circumstances, such as highly variable fitnesses or large self-weights, there can be significant differences between these outcomes for some dynamics, including BDD (but not BDB).

In this paper, we consider two dynamics where selection acts on different events. The first is BDB, a birth-death process where selection acts on the birth event, otherwise known as an Invasion Process (Lieberman et al. 2005) which has been frequently utilised in evolutionary graph theory, and adjusted to the modelling framework in Broom et al. (2015).

First, an individual $I_{i}$ is selected to reproduce with probability proportional to their fitness i.e.

3 $$\begin{aligned} b_i = \frac{F_i}{\sum _{k}F_k}. \end{aligned}$$

The offspring then replaces another individual $I_{j}$ with probability

4 $$\begin{aligned} d_{ij} = \frac{w_{ij}}{\sum _{k=1}^{N}w_{ik}}. \end{aligned}$$

The other dynamic process is BDD (Masuda 2009), where selection acts on the death event. In this evolutionary dynamic, individual $I_{i}$ is randomly selected to reproduce with probability

5 $$\begin{aligned} b_i = \frac{1}{N}. \end{aligned}$$

The offspring then replaces another individual $I_{j}$ with probability

6 $$\begin{aligned} d_{ij} = \frac{w_{ij}F^{-1}_{j}}{\sum _{k=1}^{N}w_{ik}F^{-1}_{k}}. \end{aligned}$$

The replacement weights within this paper are based on the assumption that an offspring of individual $I_{i}$ will replace individual $I_{j}$ with probability proportional to the time $I_{i}$ and $I_{j}$ spend together. The offspring of $I_{i}$ can also replace its parent $I_{i}$, and it does so with probability proportional to the time $I_{i}$ spends on its own. When $i \ne j$ The probability of individuals $I_{i}$ and $I_{j}$ meeting is given by summing all $\chi (m,\mathcal {G})$ over all *m* such that $I_{i}, I_{j} \in G$. We assume that $I_{i}$ spends an equal amount of time with all other members of group $\mathcal {G}$, therefore we weight by $1/(|\mathcal {G}| - 1)$ as there are $|\mathcal {G}| - 1$ other members of the group. However, when $i = j$, we sum $\chi (m,\mathcal {G})$ over all *m* such that $\mathcal {G} = \{i\}$. Here there is no need to weight $\chi (m,\mathcal {G})$ because $I_{i}$ is alone. The replacements weights are thus given as

7 $$\begin{aligned} w_{ij} = {\left\{ \begin{array}{ll} \sum \limits _{m}\sum \limits _{\begin{array}{c} G \\ i,j \in G \end{array}}\frac{\chi (m,G)}{|G|-1}, & i \ne j,\\ \sum \limits _{m}\chi (m, \{i\}), & i = j. \end{array}\right. } \end{aligned}$$

As our work in this paper is only focused on complete graphs, $d_{ij}$ is the same for all individuals, as all individuals are equally likely to be replaced i.e. we can simply write $d_{ij}$ as *d* (and sometimes as $d_{N}$, when we consider the influence of varying population size on *d*, since *d* depends upon *N*).

### The fixation probability

To determine the likelihood of the evolutionary success of a particular strategy within a finite population, we calculate its fixation probability. The fixation probability is regarded as the most significant quantity of a finite evolutionary process. From Broom et al. (2015), the fixation probability of a type *A* individual is defined by the following recurrence relation

8 $$\begin{aligned} p_{S}^{A} = \sum \limits _{S' \subset \{1,2,...,N\}}P_{SS'}p_{S'}^{A}, \end{aligned}$$

with boundary conditions

9 $$\begin{aligned} p_{\varnothing }^{A} = 0, \end{aligned}$$

10 $$\begin{aligned} p_{\mathcal {N}}^{A} = 1. \end{aligned}$$

Here $p_{S}^A$ is the fixation probability of a type *A* individual from the state *S*, where *S* represents the composition of the population with a certain number of type *A* individuals. $P_{SS'}$ is the probability of the population transitioning from state *S* which contains a given number of type *A* individuals. to a new state, S’ which contains a new number of type *A* individuals.

### Multiplayer games in structured populations

We used two different multiplayer games to describe the interactions between individuals. The Public Goods and Hawk–Dove games. Each of the games describes a contest between two different types of individuals, *A* and *B*. Using these games, we will describe an evolutionary process of a single type *A* individual within a population of *B*s and vice-versa to determine the fixation probability for both types of individuals.

#### The multiplayer public goods game

The multiplayer public goods game consists of two types of individuals, cooperators (*A*) and defectors (*B*). The cooperator pays a cost of *C* which is shared among the rest of the group as a reward *V* but not shared among the individual who paid the cost. Defectors pay no cost and cooperators pay a cost even when they are alone. After a game is played between a group of *a* cooperators and *b* defectors, the payoffs for a cooperator and defector are respectively

11 $$\begin{aligned} R_{a,b}^{\mathcal {A}} = {\left\{ \begin{array}{ll} R - C, & a = 1,\\ R - C + \bigg (\frac{a-1}{a+b-1}\bigg )V, & a > 1, \end{array}\right. } \end{aligned}$$

12 $$\begin{aligned} R_{a,b}^{\mathcal {B}} = {\left\{ \begin{array}{ll} R, & a = 0,\\ R + \bigg (\frac{a}{a+b-1}\bigg )V, & a > 0. \end{array}\right. } \end{aligned}$$

where *R* is the background payoffs individuals receive unrelated to the games. The public good game presented here is one of many variations with other cooperative strategy games being included in Archetti and Scheuring (2012).

#### The multiplayer Hawk–Dove game

The Hawk–Dove game was developed by Maynard Smith and Price (1973) and attempts to explain the occasional use of violence in contests over valuable resources between animals such as in populations of red deer (Clutton-Brock and Albon 1979). *A* represents the Hawk strategy, and *B* the Dove strategy. When individuals meet, they compete for a reward *V*. If all individuals in the group are Doves, then they all split the reward equally. If any Hawks are present, then the Doves concede and the Hawks fight, with the winner receiving the reward of *V* while the losing Hawks pay a cost of *C*. All individuals receive a background payoff of *R*, a reward gained from activities unrelated to the contests. In a group of *a* Hawks and *b* Doves, the average payoffs are given by

13 $$\begin{aligned} R_{a,b}^{A} = R + \frac{V - (a - 1)C}{a}, \end{aligned}$$

14 $$\begin{aligned} R_{a,b}^{B} = {\left\{ \begin{array}{ll} R, & \text { if } a > 0, \\ R + \frac{V}{b}, & \text { if } a = 0. \end{array}\right. } \end{aligned}$$

## Row-dependent movement

Row-dependent movement refers to the type of movement where the moving individual is influenced by the movement of other individuals. Various row-dependent movement mechanisms which characterise herding and dispersal behaviour were developed (Broom et al. 2020). These models serve two purposes; firstly to represent certain movement mechanisms that lead to a particular distribution of individuals over the places, and secondly to model movement distributions with certain coordinated movement properties. We will consider a target apriori distribution, denoted by $a_m$, representing the probability of a randomly selected individual going to any particular place. Our processes will be designed to achieve this target whilst moving non-independently, for example to maximise herding or dispersal. Processes where the target distribution matches the apriori distribution were called *faithful* (Broom et al. 2020).

For example, for the territorial raider model on a complete graph with *M* vertices, the apriori distribution for any individual staying at home is $\frac{h}{h+M-1}$ and moving to a specific neighbouring vertex is $\frac{1}{h+M-1}$. More generally, we can select an appropriate apriori distribution to any given movement scenario.

We first consider two movement processes where individuals are placed sequentially based on their utility functions (Broom et al. 2020). It is assumed that there is a set of utility functions $\{U_m\}$ based upon several place characteristics. The form of the utility function $U_m$ varies according to the movement distribution governing the evolutionary process. The first type of movement we consider is deterministic movement, where individuals simply move to the location which provides them with the most utility. The second is the stochastic counterpart, in the form of a polya-urn model, where an individual will have a higher probability of moving to a place that provides them with a larger utility. Then we consider a more novel type of movement, that simultaneously places all moving individuals.

### Deterministic movement: follow the majority

In this process, individual allocation to places is decided sequentially. This represents a simultaneous movement of the group, however, so that the first step of the process is to assign the ordering uniformly at random over all possible orderings (or if simulating a large population, make selection among the remaining individuals at each step of the sequence with uniform probability).

The type of deterministic movement we consider is the *follow the majority* movement process where the first individual moves to a place according to its apriori distribution and subsequent individuals simply move to the location containing the largest number of individuals. This mathematically translates to any increasing function, but the simplest example was considered (Broom et al. 2020) which we also use. The utility an individual receives from place $P_{m}$ is given by

15 $$\begin{aligned} U_{m} = Y_{m} + 1, \end{aligned}$$

where $Y_{m}$ is the current number of occupants on place $P_{m}$.

For a well-mixed process (equivalent to a territorial raider model on a complete graph with $h=1$) this leads to all individuals being in a single group, the location of which follows the apriori distribution. We note that if $h \ne 1$ we need a variant of this process to achieve the target apriori distribution, as we see in Sect. 4.1.

### Probabilistic movement: the polya-urn

Here, we consider a stochastic counterpart to follow the majority, where individuals move to a place $P_{m}$ with probability proportional to their utility function i.e. an individual moves to place $P_{m}$ with probability $U_{m}/\sum _{k}U_{k}$. This probabilistic model is represented by a standard urn model (Johnson and Kotz 1977), where balls are numbered 1, 2, ..., *M* and placed into an urn and then sequentially drawn out at random. The $n^{th}$ ball with number *m* being drawn out correspond to the $n^{th}$ individual moving to place $P_{m}$. As utility positively correlates with place occupancy, an extra ball with the same number is placed back into the urn alongside the original ball. This is represented by the following utility function

16 $$\begin{aligned} U_{m} = Ba_{m} + Y_{m}, \end{aligned}$$

where $B \in (0, \infty )$ corresponds to the initial number of balls in the urn, and $a_m$ is the apriori probability distribution. The scaling parameter *B* moderates the dependency social aggregation has on population density. $Ba_{m}$ represents the initial number of balls in the urn corresponding to place $P_{m}$. Note that as we are simply selecting the place following a probability distribution rather than actually picking out balls, there is no requirement for this number to be integer-valued.

### The wheel and base model

**Fig. 3:**
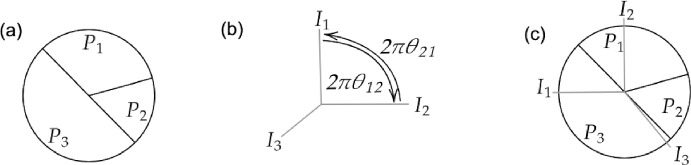
**a** $M = 3$ places with $a_1 = \frac{1}{3}, a_2 = \frac{1}{6}, a_3 = \frac{1}{2}$. **b** Represents the $N = 3$ individuals as spikes. The angle between individuals $I_{i}$ and $I_{j}$ is given by $2\pi \theta _{ij}$. In this case, $\theta _{12} = \frac{1}{4} = -\theta _{21}$. **c** Shows the simultaneous placement of all individuals after the upper disc is spun on top of the base. In this case, individuals $I_1, I_2, I_3$ move to places $P_3, P_1, P_3$ respectively

Whereas in the previous sections, an underlying movement mechanism had sequentially allocated individuals onto the places, the wheel and base model assumes a simultaneous allocation of all individuals partaking in the movement process. We suppose a base disc of perimeter 1 is divided into *M* place $P_{1},...,P_{M}$ in the shape of wedges where $P_{m}$ has perimeter length $a_{m}$ (see Fig. 3a) such that $\sum _{m}a_{m} = 1$. On top of the base disc, is an upper disc, the wheel representing the *N* individuals in the form of *N* spikes; see Fig. 3b. The angle between individuals $I_i$ and $I_j$ is given by $2\pi \theta _{ij}$, where $\theta _{ij} \in [-1/2, 1/2]$ can possibly be determined via a probability distribution. Note that $\theta _{ij} = -\theta _{ji}$. When the angles between the spikes have been set, the wheel is spun and rotates by an angle of $\theta$ selected uniformly at random. Then, individual $I_i$ moves to place $P_m$ if and only if the corresponding spike lands above the corresponding segment; see Fig. 3c.

## Theoretical results

In this section, we consider our theoretical results. Initially, we describe a generalised movement method that ensures we can achieve our apriori target for $h \ne 1$. We then consider explicit fixation probability formulae for specific cases.

### A generalised movement modelling approach

Our analysis aims to extend the existing territorial raider model to include other types of movement distributions whilst ensuring the other constituent parts of the model remain the same, that is, the population structure, the games played, and the evolutionary dynamics. By considering the home fidelity parameter and the number of connections an individual has on a complete graph, we can develop a general procedure that allows us to embed any of the considered row-dependent movement models into the evolutionary setting of the territorial raider model on complete networks. In the following, we describe a method of combining a movement process of the type described in Sect. 3 (which we refer to as following the process) with a simple additional process to achieve our apriori targets.

The procedure involves deriving a probability distribution that accounts for the various movement choices available to individuals within the population. This includes both those who follow the process and those who do not, with the available actions for the latter group depending on the value of *h*. Specifically, if $h > 1$, this indicates a preference for remaining at home; $h = 1$ represents an indifference between an individual’s home vertex and their neighbouring vertices; and $h < 1$ shows a preference for moving elsewhere. We incorporated these scenarios within the probability distribution.

Consider a complete graph where there are *M* places.

- If $h > 1$, then an individual can either partake in the process and move via the movement mechanism with probability $\frac{M}{h+M-1}$ or they do not move and stay at their home vertex with probability $\frac{h-1}{h+M-1}$.
- If $h = 1$, then every member of the population plays the process.
- If ${h} < 1$, then an individual can either move via the process with probability $\frac{Mh}{h+M-1}$ or they move to a random non-home place with probability $\frac{(M-1)(1-h)}{h+M-1}$.

Incorporating this probability distribution into the model ensures that all individuals within the population achieve the target distribution. We show how this distribution explicitly satisfies the apriori targets. If $h > 1$, the probability of an individual occupying their home vertex is $\frac{h-1}{h+M-1} + \frac{1}{M}(\frac{M}{h+M-1}) = \frac{h}{h+M-1}$ and the probability of an individual being elsewhere is $\frac{M}{h+M-1} - \frac{1}{M}(\frac{M}{h+M-1}) = \frac{M-1}{h+M-1}$. If $h < 1$, the probability of an individual occupying their home vertex is $\frac{1}{M}(\frac{Mh}{h+M-1}) = \frac{h}{h+M-1}$ and the probability of an individual being elsewhere is $\frac{(M-1)(1-h)}{h+M-1} + (\frac{Mh}{h+M-1} - \frac{1}{M}\frac{Mh}{h+M-1}) = \frac{M-1}{h+M-1}$.

As opposed to the wheel which simultaneously allocates all individuals participating in the movement process, ensuring the apriori targets are hit, sequential movement processes such as the polya-urn involve individuals moving later on in the process being influenced by preceding individuals. Assuming all individuals have the same distribution, it was proven that polya-urn process achieves the apriori targets (Broom et al. 2020), therefore this property naturally extends to our movement modelling approach. It is important to note that individuals who move via the movement mechanism are not influenced by the presence of individuals who did not move via the mechanism. This condition was important to add to our approach as it ensures the apriori targets are met. For example, an individual who moves via follow the majority, will not follow those who did not partake in the movement process. They may end up in the same place, but this will not be due to the movement mechanism process.

Regardless of the movement distribution chosen for the evolutionary model, we define a standard practice to follow when computing the fitnesses of mutants and residents within a well-mixed population, which can be characterised as follows: First, outline the distribution that describes all conceivable ways in which members of a given population can move. For each specific movement case, establish the distribution that defines all potential groupings that can emerge as a result of the considered movement case. Then, average the payoffs from each case to obtain the average payoffs. These average payoffs are used to compute the necessary evolutionary metrics such as the fitnesses for deriving an analytical expression for the fixation probability.

As an example, we examined a well-mixed population of three individuals on a complete triangle graph (see Fig. 2). Using the methodology developed in Sect. 4.1, we calculated average group distributions for each of the movement mechanisms. For $h > 1$, we show an example of the average group distribution for the follow the majority process (the polya-urn and the wheel can be found in the appendix).

- P(all together) $$= \frac{27 + 9(h-1)}{(h+2)^3},$$
- P($I_1$ $I_2$ together, $I_3$ alone) $=$ P($I_1$ $I_3$ together, $I_2$ alone) $=$ P($I_2$ $I_3$ together, $I_1$ alone) $= \frac{2(h-1)^2 + 6(h-1)}{(h+2)^3},$
- P(All individuals are alone) $= \frac{3(h-1)^2 + (h-1)^3}{(h+2)^3}.$

#### Fitness calculations

In our analysis, we evaluated the fitness of cooperators and defectors for any row-dependent movement distribution by considering the following scenario: in an *N*-sized, well-mixed population consisting of *k* cooperators and $N-k$ defectors, what proportion of reward *V* does a specific cooperator, denoted as $C_1$ receive on average?

First, we examined what fraction of *V* that $C_1$ receives from another cooperator in the population, denoted as $C_2$. We considered all possible groupings in which $C_1$ and $C_2$ could be together. We arbitrarily stated that the probability of $C_1$ and $C_2$ being together in a specific group with *S* others is $\gamma _{S+2}$. Therefore, $C_1$ receives precisely $\frac{1}{S+1}V$ from $C_2$ which is then weighted by the probability of the group forming, resulting in $V \frac{\gamma _{S+2}}{S+1}$. This quantity is then summed to consider all possible group sizes i.e. $V \sum _{S=0}^{N-2}\frac{\gamma _{S+2}}{S+1}$. This expression represents the total probability of $C_1$ and $C_2$ being in the same group, which is also a measure of how likely they are to interact, therefore, this was re-expressed as $\sum _{S=0}^{N-2}\frac{\gamma _{S+2}}{S+1} = d_N$. In other words, the total proportion of *V* that $C_1$ receives from $C_2$ can be expressed as $d_{N}V$.

17 $$\begin{aligned} F_{C} = R - C + (k-1)Vd_{N} \quad \text {and}\quad F_{D} = R + kVd_{N}. \end{aligned}$$

(17) expresses the fitness of a cooperator and defector for any movement mechanism described in Sect. 3, captured by the $d_N$ term. The value of $d_{N}$, measures the likelihood of two individuals being in the same group, thus influencing their chances of receiving rewards from each other.

A similar, more complex calculation for fitnesses in the Hawk–Dove game is provided in the appendix, assuming only independent movement for simplicity. In an *N*-sized, well-mixed population with *k* doves and $N-k$ hawks, the fitnesses for the Dove and Hawk are given by

18 $$\begin{aligned} R + \tau (h, N, k) V, \end{aligned}$$

where

$$\begin{aligned} &  \tau (h, N, k) = \left( \left( \frac{h+N-2}{h+N-1}\right) ^{N-k} - \left( \frac{(h+N-2)^{N-1}}{(h+N-1)^{N}}\right) \right. \\ &  \left. \quad \ \left( \frac{k(N-1)+(N-k)(N-1)}{k}\right) + \frac{(N-k)(N-1)(h+N-2)^{N-k-1}}{k(h+N-1)^{N-k}}\right) , \end{aligned}$$

and

19 $$\begin{aligned} R + \omega (h, N, k) V - \nu (h, N, k) C, \end{aligned}$$

where

$$\begin{aligned} \omega (h, N, k)= &  {\bigg (1 + \frac{k}{N-k} - \frac{(N-1)(h+N-2)^{N-k-1}}{(h+N-1)^{N-k}} - \frac{k(h+N-2)^{N-k}}{(N-k)(h+N-1)^{N-k}}\bigg ),} \\ \nu (h, N, k)= &  \left( \frac{k-N+1}{h+N-1} - \frac{k}{N-k} + \frac{h(N-k-1)+(N-k-1)(N-1)}{(h+N-1)^{2}} \right. \\ &  \left. + \frac{k(h+N-2)^{N-k}}{(N-k)(h+N-1)^{N-k}} + \frac{(N-1)(h+N-2)^{N-k-1}}{(h+N-1)^{N-k}}\right) . \end{aligned}$$

(18) and (19) are the Dove’s and Hawk’s fitness respectively and the calculations for these can be found in the appendix given by (41) and (42).

### General fixation probability formulae

In this section, we consider only well-mixed populations, equivalent to a complete graph with $N=M$ on a territorial raider model. The fixation probability of a mutant ($\mathcal {M}$) in an *N*-sized, well-mixed population can be expressed by the standard formula (Karlin and Taylor 1975).

20 $$\begin{aligned} \rho _{1}^{\mathcal {M}} = \frac{1}{1 + \sum \nolimits _{j=1}^{N-1}\prod \nolimits _{k=1}^{j}\frac{\delta _{k}}{\beta _{k}}}. \end{aligned}$$

Here $\beta _{K}$ and $\delta _{K}$ are the respective birth and death rates of the mutant, the ratio of which we show to be equivalent to the fitnesses of the mutant and resident respectively under BDB dynamics. The birth rate of a mutant corresponds to an offspring of the mutant replacing a resident member of the population and vice-versa for the death rate. This mathematically translates to the following equation where there are *k* mutants ($\mathcal {M}$) and $N-k$ residents ($\mathcal {R}$).

21 $$\begin{aligned} \frac{\delta _k}{\beta _k}= &  \frac{\text {P(a resident replaces a mutant)}}{\text {P(a mutant replaces a resident)}} \end{aligned}$$

22 $$\begin{aligned}= &  \frac{\frac{F_{\mathcal {R}}(dk(N-k))}{kF_{\mathcal {M}} +(N-k)F_{R}}}{\frac{F_{\mathcal {M}}(dk(N-k))}{kF_{\mathcal {M}}+(N-k)F_{R}}} \end{aligned}$$

23 $$\begin{aligned}= &  \frac{F_\mathcal {R}}{F_\mathcal {M}}. \end{aligned}$$

(20) now becomes

24 $$\begin{aligned} \rho _{1}^{\mathcal {M}} = \frac{1}{1 +\sum \nolimits _{j=1}^{N-1}\prod \nolimits _{k=1}^{j}\frac{F_{\mathcal {R}}}{F_{\mathcal {M}}}}. \end{aligned}$$

This result means that under a complete graph and BDB dynamics, for any particular game, we need only substitute the average fitnesses of the mutant and resident to determine the fixation probability. Using a similar approach, if there are *k* individuals in the set of mutants $\mathbb {K}$, and $N-k$ in the set of residents $\mathbb {L}$ the corresponding ratio of the death and birth rates under BDD is

$$\begin{aligned} \frac{\delta _k}{\beta _k}= &  \frac{\text {P(a resident replaces a mutant)}}{\text {P(a mutant replaces a resident)}}\\= &  \frac{\bigg (\frac{\frac{1}{N}w_{ij}F^{-1}_{\mathcal {M}}(k(N-k))}{\sum _{z \in \mathbb {K}} w_{iz}F^{-1}_{\mathcal {M}}+\sum _{z \in \mathbb {L} } w_{iz}F^{-1}_{\mathcal {R}}}\bigg )}{\bigg (\frac{\frac{1}{N}w_{ji}F^{-1}_{\mathcal {R}}(k(N-k))}{\sum _{z \in \mathbb {K}} w_{jz}F^{-1}_{\mathcal {M}}+\sum _{z \in \mathbb {L}} w_{jz}F^{-1}_{\mathcal {R}}}\bigg )} \\= &  \frac{\bigg (N-k+(k+w^{*})\frac{F_{\mathcal {R}}}{F_{\mathcal {M}}}\bigg )}{\bigg (k+(N-k+w^{*})\frac{F_{\mathcal {M}}}{F_{\mathcal {R}}}\bigg )}, \end{aligned}$$

where $$w = w_{ij} = w_{ji}$$, $$w_{s} = w_{ii} = w_{jj}$$ and $$w^{*} = \frac{w_{s} - w}{w}$$.

Therefore, under BDD dynamics, the fixation probability of a single mutant (20) is expressed as

25 $$\begin{aligned} \rho _{1}^{\mathcal {M}} = \frac{1}{1 + \sum \nolimits _{j=1}^{N-1}\prod \nolimits _{k=1}^{j}\frac{(N-k+(k+w^{*})\frac{F_{\mathcal {R}}}{F_{\mathcal {M}}})}{(k+(N-k+w^{*})\frac{F_{\mathcal {M}}}{F_{\mathcal {R}}})}}. \end{aligned}$$

With the fitnesses calculated, we can directly substitute them into the fixation probability of a mutant on a complete *N*-sized network under BDB dynamics (24) and BDD dynamics (25). By substituting (17) and (18) into (24) respectively, we have that the fixation probability of a mutant cooperator and dove under BDB dynamics are respectively given by

26 $$\begin{aligned} \rho _{1}^{A} = \frac{1}{1 + \sum \nolimits _{j=1}^{N-1}\prod \nolimits _{k=1}^{j}\frac{R + kVd_{N}}{R - C + (k-1)Vd_{N}}}, \end{aligned}$$

27 $$\begin{aligned} \rho _{1}^{B} = \frac{1}{1 + \sum \nolimits _{j=1}^{N-1}\prod \nolimits _{k=1}^{j}\frac{R + \omega V - \nu C}{R + \tau V}}. \end{aligned}$$

Similarly, by substituting (17) and (18) into (25), the fixation probability of a mutant cooperator and dove, under BDD dynamics are respectively given by

28 $$\begin{aligned} &  \rho _{1}^{\mathcal {A}} = \frac{1}{1 + \sum \nolimits _{j=1}^{N-1}\prod \nolimits _{k=1}^{j}\frac{(N-k+(k+w^{*})\frac{R + kVd_{N}}{R - C + (k-1)Vd_{N}})}{(k+(N-k+w^{*})\frac{R - C + (k-1)Vd_{N}}{R + kVd_{N}})}}, \end{aligned}$$

29 $$\begin{aligned} &  \rho _{1}^{\mathcal {B}} = \frac{1}{1 + \sum \nolimits _{j=1}^{N-1}\prod \nolimits _{k=1}^{j}\frac{(N-k+(k+w^{*})\frac{R + \omega V - \nu C}{R + \tau V})}{(k+(N-k+w^{*})\frac{R + \tau V}{R + \omega V - \nu C})}}. \end{aligned}$$

### Weak selection

The concept of selection intensity to consider situations in which the game exerts a minor influence on the evolutionary process was considered and the *rule of 1/3* was established (Taylor et al. 2004) and states that selection favours type *A* fixating if the internal equilibrium point is less than 1/3. This general rule was considered for the Hawk–Dove game and it was found that if $\frac{V}{C} > \frac{2}{3}$, then selection favours the fixation of the dove. It is worth noting that this analysis only considered pairwise contests between individuals therefore, we have extended this analysis to encompass the multiplayer Hawk–Dove game from our model, allowing us to explore the effects multiplayer interactions have on the evolution of cooperation. We considered the effect weak selection has on the fixation formulae in Sect. 4.2 by assuming *R* is very large compared to *V* and *C* i.e. the game has little influence in the evolutionary process.

#### The public goods game

We first considered the cooperator’s fixation probability under BDB. Consider the expression inside the product term of (26).

30 $$\begin{aligned} \frac{R + kVd_{N}}{R - C + (k-1)Vd_{N}}&\approxeq&1 + \frac{Vd_{N} + C}{R}, \end{aligned}$$

so (26) now becomes

31 $$\begin{aligned} \frac{1}{1 + \sum _{j = 1}^{N - 1}(1 + \frac{Vd_{N} + C}{R})^{j}}. \end{aligned}$$

The term inside the summation can be approximated by the following,

32 $$\begin{aligned} \bigg (1 + \frac{Vd_{N} + C}{R}\bigg )^{j} \approxeq 1 + j\bigg (\frac{Vd_{N} + C}{R}\bigg ). \end{aligned}$$

Therefore, (31) becomes

33 $$\begin{aligned} \frac{1}{1 + \sum _{j = 1}^{N - 1}(1 + j(\frac{Vd_{N} + C}{R}))}, \end{aligned}$$

which simplifies to

34 $$\begin{aligned} \frac{1}{N + (\frac{Vd_{N} + C}{R})\sum _{j = 1}^{N - 1}(j)}= &  \frac{1}{N}\bigg (\frac{1}{1+\frac{N-1}{2R}(Vd_{N}+C)}\bigg ) \nonumber \\&\approxeq&\frac{1}{N}\bigg (1 - \frac{N-1}{2R}(Vd_{N}+C)\bigg ). \end{aligned}$$

From (34), as the parameter $d_{N}$ increases, the situation becomes increasingly unfavourable for the mutant cooperator due to the defector’s advantageous position. The defector can receive an additional reward without incurring any cost because, from their perspective, there is an extra cooperator within the population from whom they will receive this benefit. Conversely, the cooperator does not have this advantage as they receive no share from their own contributions. With the growing value of $d_N$, the likelihood of the mutant cooperator interacting with defectors rises, further reinforcing the defector’s advantageous position.

We also considered the cooperator’s fixation probability under BDD dynamics. By applying similar weak selection methods to (28), we have

35 $$\begin{aligned} \frac{1}{N}\bigg (1 - \frac{(N+2w^{*})(N-1)}{2R(N+w^{*})}(Vd_{N}+C)\bigg ). \end{aligned}$$

(35) is an approximation of the fixation probability of the mutant cooperator under BDD dynamics.

#### The Hawk–Dove game

We carried out a similar, more complicated calculation for considering the dove’s fixation probability which can be found in the appendix. Using the dove’s fixation probability (27), a calculation was done to determine the dove’s neutrality condition by setting the dove’s fixation probability to equal $\frac{1}{N}$ i.e. $\rho ^{B}_{1} = \frac{1}{N}$.

36 $$\begin{aligned} V = \frac{(\frac{1}{2}-\frac{1}{\textrm{e}})}{(\frac{1}{\textrm{e}}(\gamma - 1 - f(h)) + 1)}C, \end{aligned}$$

where $$f(h) = H[N-1, \bigg (\frac{h+N-1}{h+N-2}\bigg )^{k}] - \ln (N-1)$$ and $$H[N-1, a] = \sum _{k=1}^{N-1}\frac{a^{k}}{k}$$

For varying *h*, the neutrality condition is approximately given by $C = 1.11 V$ which means that under our models, hawks are generally worse off compared to doves as the cost does not need to be raised as significantly in the classical models for hawks and doves to be doing equally well. This intuitively makes sense as larger groups are generally bad for hawks who are more likely to encounter competition and, therefore, incur a greater cost due to a larger presence of other hawks in their game interactions. We also applied weak selection methods to the dove’s fixation probability under BDD dynamics which can be found in the appendix. We saw that the dynamics do not affect the dove’s neutrality condition.

The BDD approximations for the fixation probabilities of the cooperator (35) and dove (64) have a similar form to their respective BDB approximations (34), (51). If $w^{*} = 0$, then the approximations are equal. In other words, if the self-weights are equal to all other weights, then under weak selection, the fixation probability of a mutant cooperator or dove is the same regardless of whether selection acts on the first or second event. Other dynamics were considered and their functionality was explained in Pattni et al. (2017), such as the DBD dynamics where death acts first and selection acts on this event. It was found that the results of DBD and BDB were identical. If the self-weights are the same as all other weights, then implementing DBD is equivalent to BDD; therefore, BDD is the same as BDB.

A general condition for the fixation probability of a type *A* mutant in a type *B* population is greater than the fixation probability of a type *B* mutant in a type *A* population was established in Tarnita et al. (2009) given by

37 $$\begin{aligned} \sigma a + b > c + \sigma d. \end{aligned}$$

where $\sigma$ is the *structure coefficient* of the process. The value of $\sigma$ depends on both the graph and the updating rule, but not on the values *a*, *b*, *c* and *d* (which are the payoffs to the pairwise matrix game) for example. For regular graphs with degree *k* and $N \gg k$, we have $\sigma = \frac{k+1}{k-1}$. Using this analysis for the pairwise Hawk–Dove game, it was shown that in an infinite, well-mixed population $(k \rightarrow \infty )$, hawks and doves do equally well when $V = 2C$. We also extended this analysis to our models under the assumption of an infinite, well-mixed population, where hawks and doves interact with one another in arbitrary group sizes rather than limiting pairwise interactions.

By considering the fitness of a dove and hawk in an infinite, well-mixed population with a proportion of *p* doves, we were able to extend the analysis from Tarnita et al. (2009) by introducing a multiplayer Hawk–Dove game. By using the substitution $p = \frac{k}{N}$, and then assuming $N \rightarrow \infty$, the fitnesses of a dove (18) and hawk (19) are respectively given by

38 $$\begin{aligned} &  R + \bigg (\frac{e^{p} - 1}{ep}\bigg )V, \end{aligned}$$

39 $$\begin{aligned} &  R + \bigg (\frac{1 - e^{p-1}}{1-p}\bigg )V - \bigg (\frac{e^{p-1} - p}{1-p}\bigg )C. \end{aligned}$$

By equating these two fitnesses together and solving for $\frac{V}{C}$, we have

40 $$\begin{aligned} \frac{V}{C} = \frac{ep(e^{p-1}-p)}{(1-e^{p-1})(ep)-(e^{p}-1)(1-p)}. \end{aligned}$$

For each value of *p*, (40) provides the corresponding equilibrium ratio of $\frac{V}{C}$. Our point of interest is at $p = \frac{1}{2}$ where both doves and hawks are doing equally well. This equilibrium condition is given by $\frac{V}{C} = 0.688$ i.e. $C = 1.453 V$ which supports our previous neutrality condition for a dove (36), that in a multiplayer game context, hawks are generally doing worse than in the traditional pairwise game analysis.

## Numerical results

For considering higher populations on larger graphs, we carried out computational methods to simulate such processes as analytically carrying them out would be impractical. The computational methods are the same as the ones carried out in Schimit et al. (2019) except here, the simulations are carried out on much simpler, complete networks, and individuals move via our approach developed in Sect. 4.1.

One simulation is defined as follows:

- The chosen complete network is formed using the iGraph library (Csardi and Nepusz 2006).
- The mutant is randomly placed on one of the nodes.
- Every individual probabilistically moves (or not) from their home vertex according to the parameters of the model. Groups are formed and multiplayer games are played where $R = 10, C = 1$ and $V = 2$ for both of the considered games.
- Individuals return to their home patches.
- Each individual moves (or not) and groups are formed. Here, no games are played, instead, the dynamic process occurs. One individual is selected to reproduce an offspring that will replace another random member of the group (or its parent if the parent is alone). Selection either acts on the birth or death even according to the chosen dynamics.
- The simulation terminates once the population is entirely composed of a single type of individual.
- This process is averaged over 1,000,000 runs to minimise statistical variability.

As discussed in Sect. 2.3, the assumptions in this section are slightly different to Sect. 4. In the simulations, a single step is used in the contests and in the dynamic process i.e. individuals only move once. The theoretical section assumes average weights corresponding to where individuals move many times to accrue average fitnesses and weights.

**Fig. 4:**
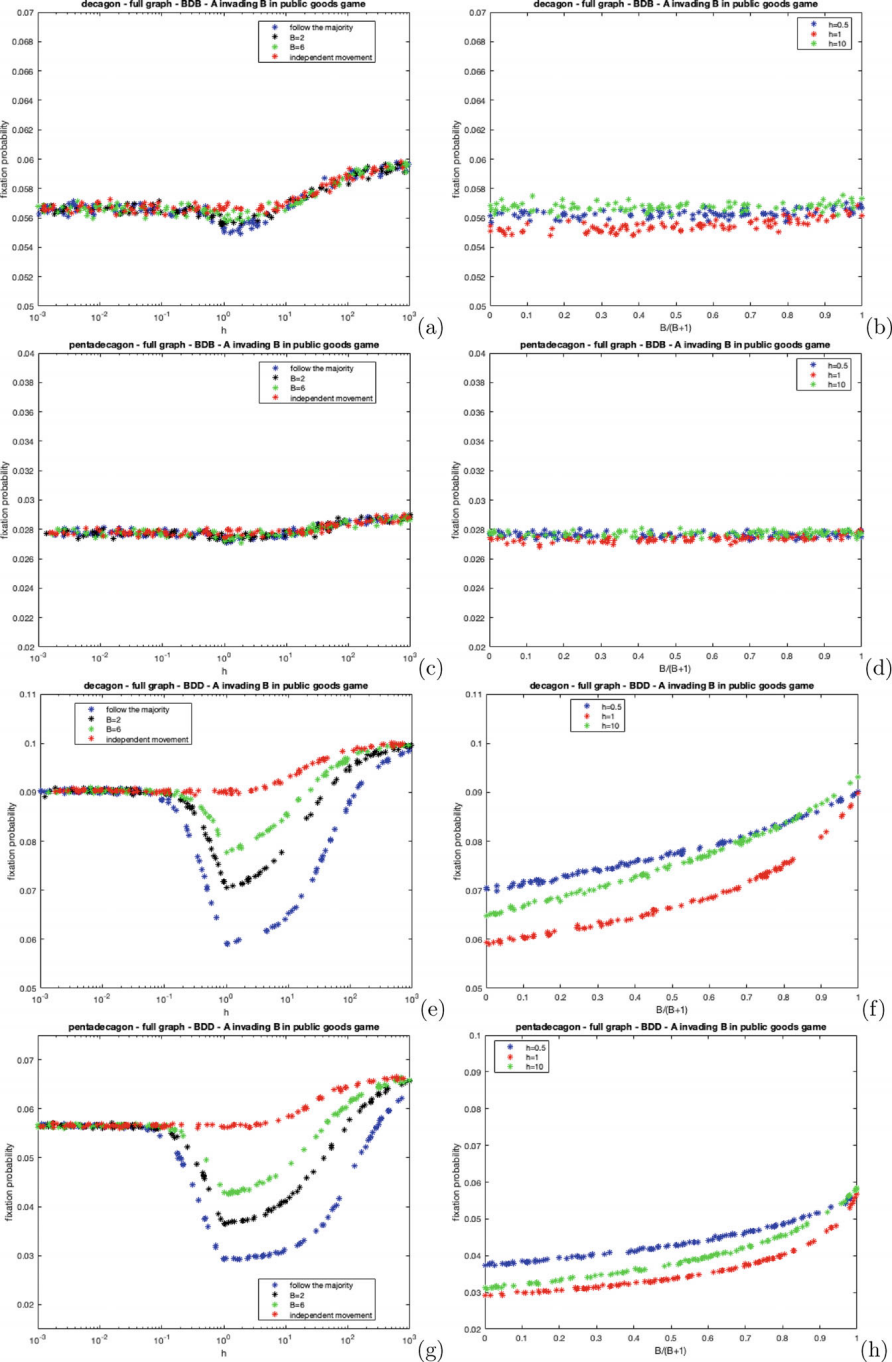
The fixation probability of a mutant cooperator in a population of defectors on complete decagon and pentadecagon graphs under BDB and BDD dynamics for varying *h* under distinct polya-urn movement processes, For **a**, **c**, **e** and **g**, we set $B = 0$ (follow the majority), $B = 2$, $B = 6$ and $B = 10{,}000$ (a sufficiently large value to mirror independent movement). For **b**, **d**, **f** and **h** we set $h = 0.5$, $h = 1$, and $h = 10$ and vary *B*

Figure 4 illustrates the fixation probability of a mutant cooperator under polya-urn processes for BDB and BDD dynamics on complete decagon and pentadecagon graphs. As *h* approaches 0.1, the fixation probability remains constant, attributed to individuals randomly moving to non-home places.

The cooperator’s fixation probability reaches its lowest point when $h = 1$ where all members of the population must participate in the movement process, leading to the formation of groups of varying sizes (depending on the type of movement governing the process). This is disadvantageous for cooperators as they are more likely to encounter defectors. The "follow the majority" process is the worst type of movement for cooperators as it ensures all individuals partaking in the movement process, herd together at the same place; therefore, ensuring that defectors receive rewards from cooperators. As *h* tends to larger values, regardless of the movement process, the cooperator’s fixation probability gradually increases because individuals are more likely to remain on their own therefore, cooperators are highly unlikely to interact with defectors, thus increasing their relative fitness.

Figure 4 also shows plots of the fixation probability of the cooperator against *B* (scaled to $\frac{B}{B+1}$). As *B* increases, the cooperator’s fixation probability increases. This is attributed to the gradual shift in the movement mechanism from a deterministic type ($B = 0$), where individuals simply move to the place containing the largest number of individuals, to an independent type ($B \rightarrow \infty$) where individuals move randomly, without influence from other individuals. As *B* increases, individuals are less likely to herd together therefore the relative difference in the average cooperator’s and defector’s fitness gradually decreases, thus increasing the cooperator’s fixation probability.

The cooperator’s fixation probability is higher under BDD dynamics because selection affects the second event. During the birth event, the probability of the cooperator reproducing is simply $\frac{1}{N}$ (5) as opposed to the less favourable BDB dynamics where the probability is proportional to the cooperator’s fitness (3). For large *h*, the fixation probability tends to $\frac{1}{N}$ shown in Fig. 4e, g. Here, individuals are mostly alone or occasionally with another individual. If an alone individual is randomly selected to reproduce, then its offspring will replace them. Suppose an individual within a pair is randomly selected to reproduce. In that case, the other individual within the pair is guaranteed to be replaced, thus rendering the influence of selection within the replacement process irrelevant.

Furthermore, Fig. 4 shows that row-dependent movement has a more prominent effect on the cooperator’s fixation probability when selection acts on the second event. In Fig. 4e–h, there is a greater disparity in the fixation probabilities between the different movement processes compared to Fig. 4a–d where there is a smaller effect. Under BDD dynamics, even though cooperators are more likely to reproduce, they are also more likely to be replaced (depending on the movement mechanism governing the process). For instance, if individuals are moving via follow the majority and $h = 1$, then all individuals herd together and cooperators are more likely to be replaced because of selection acting on the replacement event (6). Whereas under BDB dynamics, all individuals within the group are equally likely to be replaced.

**Fig. 5:**
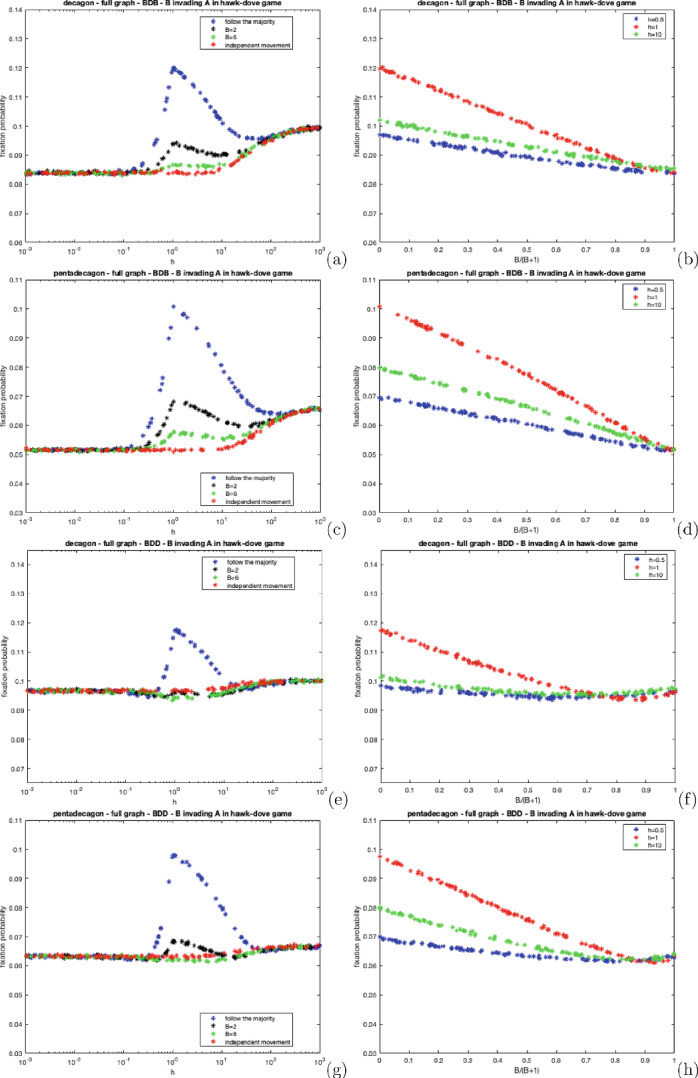
The fixation probability of a mutant dove in a population of hawks on complete decagon and pentadecagon graphs under BDB and BDD dynamics for varying *h* under distinct polya-urn movement processes, For **a**, **c**, **e** and **g**, we set $B = 0$ (follow the majority), $B = 2$, $B = 6$ and $B = 10{,}000$ (a sufficiently large value to mirror independent movement). For **b**, **d**, **f** and **h** we set $h = 0.5$, $h = 1$ and $h = 10$ and vary *B*

Figure 5 portrays the fixation probability of a mutant dove under distinct polya-urn processes for BDB and BDD dynamics on the complete decagon and pentadecagon. In these figures, as *h* approaches one, the dove’s fixation probability increases and reaches its maximum when $h = 1$.

As all members of the population partake in the movement process when $h = 1$, hawks are more likely to interact with one another, incurring greater costs, thus reducing their relative fitness. Therefore, in this game, follow the majority is the most beneficial movement process for doves because this process forces all hawks partaking in the movement process to interact with each other. As *h* increases, the dove’s fixation probability decreases because hawks are more likely to stay on their home vertices and, therefore, less likely to interact with each other, increasing their relative fitness. As *h* becomes infinitely large, the dove’s fixation probability tends to $\frac{1}{N}$ regardless of the dynamics. Hawks and doves will have the same fitness if they are always alone therefore, selection does not affect the process. Also, Fig. 5 shows that as *B* increases, the dove’s fixation probability falls. This occurs because as *B* increases, hawks are no longer forced to group, thus their relative fitness gradually increases alongside *B*.

**Fig. 6:**
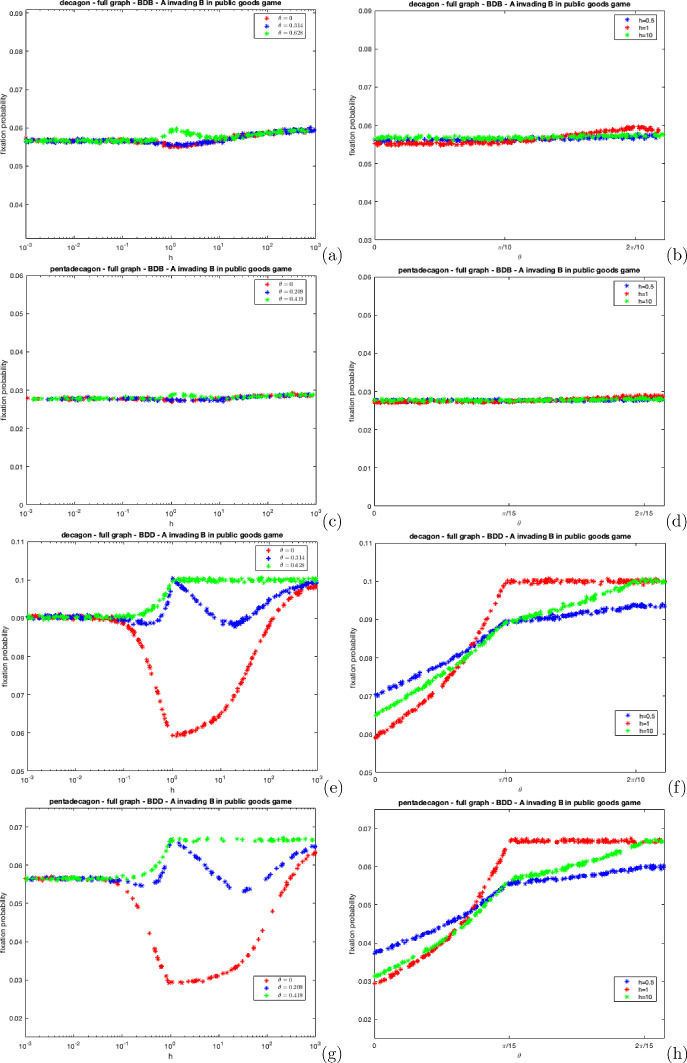
The fixation probability of a mutant cooperator in a population of defectors on complete decagon and pentadecagon graphs under BDB and BDD dynamics for varying *h* under distinct wheel movement processes, For **a**, **c**, **e** and **g**, we set $\theta = 0$ (follow the majority), $\theta = \frac{2\pi }{N}$ (represents a near complete dispersal process), $\theta = \frac{\pi }{N}$. For **b**, **d**, **f** and **h** we set $h = 0.5$, $h = 1$ and $h = 10$ and vary $\theta$

Furthermore, if selection acts on the second event, independent movement is no longer the worst type of movement for doves. Instead, a polya-urn process (close to independent movement) is the worst type of movement as shown in Fig. 5f, h, where the value of $\frac{B}{B+1}$ reaches its lowest point slightly below 1 but begins to increase after. This occurs due to the combined effects of the game and dynamics but this effect is largely insignificant.

Figure 6 shows the fixation probability of a mutant cooperator under the wheel process for both BDB and BDD dynamics on the complete decagon and pentadecagon. The chosen values of theta remain consistent for each graph. $\theta = 0$ represents the follow the majority process, while $\theta = \frac{2\pi }{N}$ signifies a near complete dispersal process where all individuals are separated. Note that in our simulations, theta is rounded to three decimal places to allow for a minimal degree of pairwise interaction between individuals under this angle. Without this adjustment, the simulation would fail to complete as individuals would only replace themselves if they were always separated, thus the evolutionary process would never reach extinction or fixation. $\theta = \frac{\pi }{N}$ corresponds to an intermediary angle between complete herding and separation.

The trends depicted in Fig. 6 resemble those observed in the polya-urn in Fig. 4, particularly concerning the influences of herding, dynamics, and the level of *h* have on the cooperator’s fixation probability. However, the key finding from these figures is that $\theta = \frac{2\pi }{N}$, provides the maximum fixation probability for the mutant cooperator for all *h*. When $h = 1$ and $\theta = \frac{2\pi }{N}$, all individuals are nearly always alone. This leads to an increase in the cooperator’s relative fitness, as they rarely provide any rewards to defectors. Consequently, the fixation probability rises significantly at this point. Figure 6e, g show that when $\theta = \frac{2\pi }{N}$ or $\theta = \frac{\pi }{N}$ and $h = 1$, the fixation probability is $\frac{1}{N}$ because individuals are either alone or in a pair rendering selection insignificant as fitness is negligible in these cases due to selection acting on the second event.

**Fig. 7:**
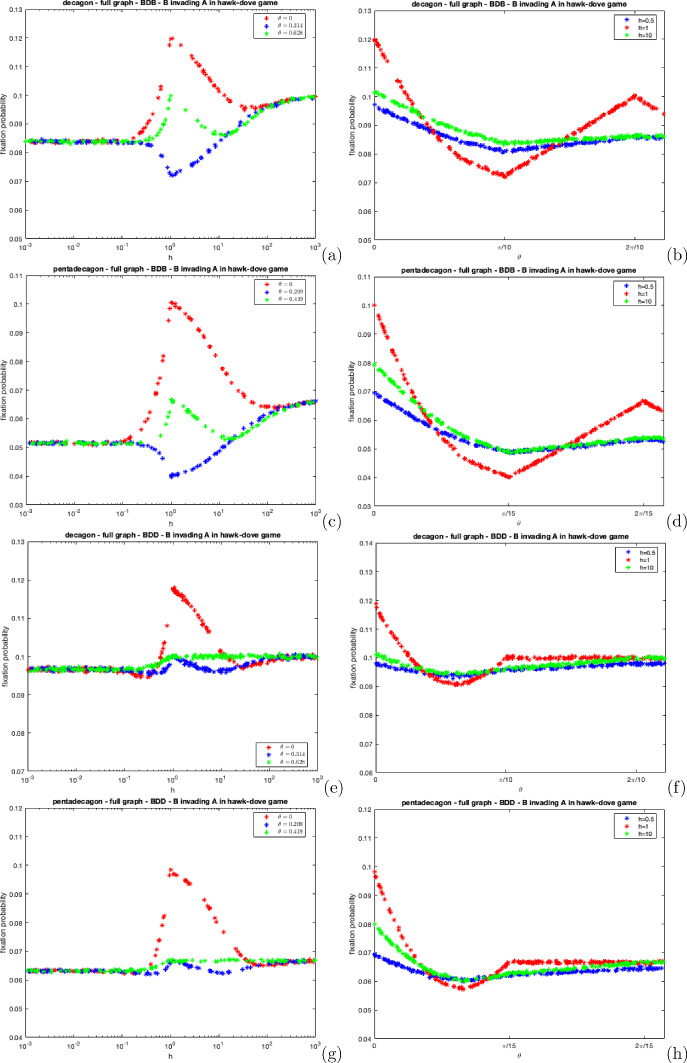
The fixation probability of a mutant dove in a population of hawks on complete decagon and pentadecagon graphs under BDB and BDD dynamics for varying *h* under distinct wheel movement processes, For **a**, **c**, **e** and **g**, we set $\theta = 0$ (follow the majority), $\theta = \frac{2\pi }{N}$ (represents a near complete dispersal process), $\theta = \frac{\pi }{N}$. For **b**, **d**, **f** and **h** we set $h = 0.5$, $h = 1$, and $h = 10$ and vary $\theta$

Figure 7 depicts the fixation probability of a mutant dove under the wheel process for both BDB and BDD dynamics on the complete decagon and pentadecagon.

Figure 7a–d show that when $h = 1$ and $\theta = \frac{2\pi }{N}$, the dove’s fixation probability is $\frac{1}{N}$ despite selection acting on the first event. This occurs as nearly every member of the population is separated, therefore individuals do not compete with each other over resources. Therefore, both hawks and doves have the same fitness rendering selection insignificant. When $h = 1$ and $\theta = \frac{\pi }{N}$, the fixation probability is at its lowest. Under this angle, there are at most pairwise groups which is beneficial for hawks who incur very small costs from the game interactions.

Also, Fig. 7 shows that follow the majority $(\theta = 0)$ gives a fixation probability greater than $\frac{1}{N}$. As there is a large native hawk population, they herd together leading to them incurring significant costs, greatly reducing their relative fitness, therefore, increasing the dove’s fixation probability. In the Hawk–Dove Game, it is clear that herding favours the evolution of cooperation more than dispersal.

Below, we show a Table 1 summarising how the different movement processes generally affect the mutant cooperator’s and dove’s fixation probability (FP).

## Discussion

In this paper, we have developed the framework from Broom and Rychtar (2012), by considering the evolution of structured populations on complete networks involving multiplayer interactions where individuals move in a coordinated manner (row-dependent movement). Specifically, we have extended the territorial raider model developed by Broom et al. (2015) as we have devised a methodology to model an evolutionary process where individuals move in a coordinated manner described by the movement mechanisms developed by Broom et al. 2020. In previous models, (Broom et al. 2015; Schimit et al. 2022) individuals moved independently irrespective of how other individuals moved. Other models (Pattni et al. 2018; Erovenko et al. 2019; Pires et al. 2023; Erovenko and Broom 2024) involved the development of a Markov movement model, where the movement of individuals depends upon the population’s history. Hence, the model in this paper provides a different perspective on the movement of individuals. In particular, we explored the relation between row-dependent movement and the evolution of cooperation.

The main objective of this paper was to embed realistic coordinated movement systems into a complete evolutionary setting and use different social dilemma games to illustrate this as this has previously not been considered in modelling the evolution of structured populations. In Krieger et al. (2017) the effects of an abstract type of motion on the evolution of cooperation in structured populations were explored. In the context of evolutionary graph theory, individuals swap or shuffle vertices on the graph structure, independent of the reproductive events. They demonstrated that the presence of motion can amplify or suppress selection depending on the graph structure. For instance, motion suppresses selection on the cycle graph. However, it was also shown that this type of motion did not change the population’s configuration on the complete graph and, therefore, has no effects on the evolutionary dynamics. This, however, differs from our results in this paper focused on complete graphs as we have illustrated the several effects the movement mechanisms have on the evolution of cooperation. However, the work done in our paper is largely different as individuals move more realistically and can form multiplayer groups.

**Table 1:** Fixation probabilities (FP) of cooperators and doves under different movement processes: follow the majority ($B=0$), polya-urn (increasing *B*), random movement ($B \rightarrow \infty$), and the wheel (separation angle)

	Cooperator’s FP	Dove’s FP
Follow the majority $(B=0)$	Minimum	Maximum
Polya-Urn (increasing *B*)	Increases	Decreases
Random movement ($B \rightarrow \infty$)	Increases	Minimum
The wheel (separation angle)	Maximum	Increases

In the context of the Public Goods Game, we demonstrated that herding hinders the evolution of cooperation as aggregation provides defectors with opportunities to exploit cooperators in their contest interactions. Dispersal, however, increases the likelihood of cooperative behaviour evolving as defectors are less likely to be in groups containing cooperators and, therefore, cannot receive a benefit from their presence. Ohtsuki et al. (2006) showed that, in general, birth-death processes do not favour the evolution of cooperation. Consequently, in the public goods game, the cooperator’s fixation probability is always under 1/*N*, even with the implementation of the movement mechanisms. However, in the Hawk–Dove Game, aggregation benefits the evolution of cooperation. In Broom et al. (2015), it was shown that the dove’s fixation probability can occasionally exceed 1/*N* if the reward is adjusted. However, the results in this paper show that even if the reward remains constant, the movement distributions, particularly follow the majority, have a stronger effect in increasing the dove’s fixation probability above 1/*N* as hawks are forced to herd together. This forces hawks to interact with one another, incurring a greater cost, thus decreasing their relative fitness. While dispersal also benefits doves, herding has a stronger effect.

Moreover, we derived analytical expressions for the fixation probabilities of the cooperator and dove in both BDB and BDD dynamics. By applying weak selection methods, we extended previous analyses (Tarnita et al. 2009; Taylor et al. 2004) by producing neutrality and equilibrium conditions for the Hawk–Dove game. These conditions align with our expectations, indicating that, in the models developed in this paper, hawks generally perform worse than in the traditional evolutionary graph theory models. The work in this paper accounts for a more realistic multiplayer game scenario compared to the limiting pairwise case. Notably, larger group sizes negatively impact the hawk’s fixation probability as expected.

There are several directions for future work. Broom et al. (2015) explored other evolutionary measures such as mean group size and temperature and their impact on fixation probability. Our primary focus was to develop a methodology to explore the relationship between row-dependent movement and fixation probabilities. We intend to investigate these evolutionary measures and their relationship with the movement mechanisms under our models in future work. Another potential direction involves extending the methodology developed in this paper to the generalised territorial raider model established by (Pattni et al. 2017). This extension would allow for the consideration of evolutionary processes where individuals reside within subpopulations and move according to the movement distributions described in this paper. We also intend to consider non-complete graph structures, representing non-well-mixed populations, where individuals will have different apriori distributions. Incorporating such graph structures into the evolutionary process raises the question of whether our developed methodology in this paper will naturally extend to the non-well-mixed case accommodating the various, distinct apriori distributions. Alternatively, it may be the case that a new approach will need to be developed. Furthermore, the movement mechanisms could also be adjusted to instead allow for biased movement dependent upon the strategies present at each of the patches. For example, the follow the majority process could be amended to state that individuals move to the patch that contains the largest number of cooperators. A much more complex avenue involves the simultaneous implementation of both row-dependent movement and history-dependent movement within the evolutionary process. In the Markov models (Pattni et al. 2018), individuals prefer to remain at places that benefit their fitness, characterised by parameters measuring an individual’s preference for staying in a specific group, such as the staying propensity and a group’s attractiveness. We aim to investigate how these parameters will need to be modified to also accommodate row-dependent movement.

## Appendix

### Average group distribution on complete triangle graph

Using the methodology defined in Sect. 4.1, we showed how to calculate the average group distribution by considering a well-mixed population of three individuals $I_1$, $I_2$, and $I_3$ on a complete triangle graph under the follow the majority, Polya-urn, and wheel processes.

The average group distribution for the follow the majority process is

- P(all individuals are together) $= \frac{9(h-1)}{(h+2)^3} + \frac{27}{(h+2)^3} = \frac{27 + 9(h-1)}{(h+2)^3}$,
- P($I_1$ and $I_2$ are together while $I_3$ is alone) $= \frac{2(h-1)^2}{(h+2)^3} + \frac{6(h-1)}{(h+2)^3} = \frac{2(h-1)^2 + 6(h-1)}{(h+2)^3}$,
- P($I_1$ and $I_3$ are together while $I_2$ is alone) $= \frac{2(h-1)^2}{(h+2)^3} + \frac{6(h-1)}{(h+2)^3} = \frac{2(h-1)^2 + 6(h-1)}{(h+2)^3}$,
- P($I_2$ and $I_3$ are together while $I_1$ is alone) $= \frac{2(h-1)^2}{(h+2)^3} + \frac{6(h-1)}{(h+2)^3} = \frac{2(h-1)^2 + 6(h-1)}{(h+2)^3}$,
- P(all individuals are alone) $= \frac{3(h-1)^2}{(h+2)^3} + \frac{(h-1)^3}{(h+2)^3} = \frac{3(h-1)^2 + (h-1)^3}{(h+2)^3}$.

The average group distribution under a general polya-urn process is given by

- P(all individuals are together) $= \frac{3\left( h-1\right) \left( B+3\right) \left( B+2\right) +3\left( B+3\right) \left( B+6\right) }{\left( h+2\right) ^{3}\left( B+1\right) \left( B+2\right) }$,
- P($I_1$ and $I_2$ are together while $I_3$ is alone) $= \frac{2\left( h-1\right) ^{2}\left( B+1\right) \left( B+2\right) +6\left( h-1\right) \left( B+3\right) \left( B+6\right) + 3\left( B+3\right) \left( 2B\right) }{\left( h+2\right) ^{3}\left( B+1\right) \left( B+2\right) }$,
- P($I_1$ and $I_3$ are together while $I_2$ is alone) $= \frac{2\left( h-1\right) ^{2}\left( B+1\right) \left( B+2\right) +6\left( h-1\right) \left( B+3\right) \left( B+6\right) + 3\left( B+3\right) \left( 2B\right) }{\left( h+2\right) ^{3}\left( B+1\right) \left( B+2\right) }$,
- P($I_2$ and $I_3$ are together while $I_1$ is alone) $= \frac{2\left( h-1\right) ^{2}\left( B+1\right) \left( B+2\right) +6\left( h-1\right) \left( B+3\right) \left( B+6\right) + 3\left( B+3\right) \left( 2B\right) }{\left( h+2\right) ^{3}\left( B+1\right) \left( B+2\right) }$,
- P(all individuals are alone) $= \frac{3\left( h-1\right) ^{2}\left( B+1\right) \left( B+2\right) +3\left( h-1\right) \left( 2B\right) \left( B+2\right) + 3B\left( 2B\right) + \left( h-1\right) ^{3}\left( B+1\right) \left( B+2\right) }{\left( h+2\right) ^{3}\left( B+1\right) \left( B+2\right) }$.

For the wheel process, we considered an example where $0\le \theta <\frac{\pi }{3}$,

- P(All individuals are together) $= \frac{9h(1-\frac{2\theta }{\pi })+18-\frac{63\theta }{\pi }}{(h+2)^3}$,
- P($I_1$ and $I_2$ are together but not with $I_3$) $=\frac{2(h-1)^2+6(h-1)+\frac{27\theta }{\pi }}{(h+2)^3}$
- P($I_1$ and $I_3$ are together but not with $I_2$) $=\frac{2(h-1)^2+6(h-1)+\frac{27\theta }{\pi }}{(h+2)^3}$,
- P($I_2$ and $I_3$ are together but not with $I_1$) $=\frac{2(h-1)^2+6(h-1)+\frac{27\theta }{\pi }}{(h+2)^3}$,
- P(All individuals are separate) $=\frac{3(h-1)^2+27(h-1)(\frac{2\theta }{3\pi })+(h-1)^3}{(h+2)^3}$.

### The fitness of a dove and hawk

In the Hawk–Dove game, we opted to assume only independent movement to simplify the fitness calculation. This simplification was necessary because the Hawk–Dove game exhibits greater complexity in the payoffs to each strategy, which are contingent on group composition and, therefore, the movement distribution. By focusing on solely independent movement for this game, we were able to evaluate the fitness of hawks and doves within this framework more effectively.

Consider a population of size *N*, well-mixed, and composed of *k* doves and $N-k$ hawks. A dove will only receive a proportion of a reward *V* if it is present in a group that contains no hawks. This can occur in four distinct scenarios. Consider two doves, $D_1$, $D_2$ and a hawk $H_1$:

- $D_1$ remains in its home, and a group of *L* doves forms on $D_1$’s home patch.
- $D_1$ moves to $D_2$’s home patch, where $D_2$ stays at home, and a group of *L* doves forms on $D_2$’s home patch.
- $D_1$ moves to $D_2$’s home patch, where an *L*-sized group of doves forms, but $D_2$ leaves their home and moves elsewhere.
- $D_1$ moves to $H_1$’s home patch, where an *L*-sized group of doves forms, but $H_1$ leaves their home and moves elsewhere.

To compute the average fitness of a dove, we weighted the reward that $D_1$ receives in each of these scenarios by the probability of each group forming. We consider the first scenario as an example. The probability of $D_1$ staying at home and an *L*-sized group of doves forming on $D_1$’s home patch is given by

$$\begin{aligned} \beta _{L} = \bigg (\frac{h}{h+N-1}\bigg )\bigg (\frac{1}{h+N-1}\bigg )^{L-1}\left( {\begin{array}{c}k-1\\ L-1\end{array}}\right) \bigg (\frac{h+N-2}{h+N-1}\bigg )^{N-k}\bigg (\frac{h+N-2}{h+N-1}\bigg )^{k-L}. \end{aligned}$$

Note that the term $\left( \frac{h+N-2}{h+N-1}\right) ^{N-k}$ ensures the absence of hawks in the group, and $\left( \frac{h+N-2}{h+N-1}\right) ^{k-L}$ ensures that all other $k-L$ doves are located elsewhere. We must then weight $\beta _{L}$ by the number of doves in the group, as each dove receives an equal share of the reward, resulting in $\beta _{L}(\frac{1}{L})V$. This is summed over all possible group sizes to cover the entire range, $\sum _{L = 1}^{k}\beta _{L}(\frac{1}{L})V$. This expression can be simplified as follows

$$\begin{aligned} \sum \limits _{L = 1}^{k}\beta _{L}(\frac{1}{L}) = \frac{h}{k}\bigg ( \bigg (\frac{h+N-2}{h+N-1}\bigg )^{N-k} - \bigg (\frac{h+N-2}{h+N-1}\bigg )^{N}\bigg ). \end{aligned}$$

By employing similar methods for the other scenarios and combining these expressions, we derive the average fitness of a dove as:

$$\begin{aligned} &  R + \left( \left( \frac{h+N-2}{h+N-1}\right) ^{N-k} - \left( \frac{(h+N-2)^{N-1}}{(h+N-1)^{N}}\right) \left( \frac{k(N-1)+(N-k)(N-1)}{k}\right) \right. \\ &  \quad \left. + \frac{(N-k)(N-1)(h+N-2)^{N-k-1}}{k(h+N-1)^{N-k}}\right) V, \end{aligned}$$

which we re-express as

41 $$\begin{aligned} R + \tau (h, N, k) V, \end{aligned}$$

where

$$\begin{aligned} &  \tau (h, N, k) = \left( \left( \frac{h+N-2}{h+N-1}\right) ^{N-k} - \left( \frac{(h+N-2)^{N-1}}{(h+N-1)^{N}}\right) \right. \\ &  \quad \left. \left( \frac{k(N-1)+(N-k)(N-1)}{k}\right) + \frac{(N-k)(N-1)(h+N-2)^{N-k-1}}{k(h+N-1)^{N-k}}\right) . \end{aligned}$$

Similarly, to calculate the fitness of a hawk, we must consider all scenarios in which a hawk can receive a share of the reward and possibly endure a cost. Hawks are indifferent to the presence of doves within the group, as they will always flee from a hawk’s presence, leading to them receiving no share of the reward. The portion of *V* that a hawk receives depends on whether other hawks are present within the group. Consider two hawks, $H_1$ and $H_2$, along with a dove, $D_1$:

- $H_1$ stays at home, and a group of *L* hawks forms on $H_1$’s home patch.
- $H_1$ moves to $D_1$’s home patch, where a group of *L* hawks forms.
- $H_1$ moves to $H_2$’s home patch, where $H_2$ stays home and a group of *L* hawks is formed.
- $H_1$ moves to $H_2$’s home patch, where a group of *L* hawks forms, but $H_2$ has moved elsewhere.

To calculate the average fitness of a hawk, we must weight the reward that $H_1$ receives in each of these scenarios by the probability of each group forming. Consider the first scenario as an example. The probability of $H_1$ staying at home and a group of *L* hawks forming on $H_1$’s home patch is given by

$$\begin{aligned} \alpha _{L} = \bigg (\frac{h}{h+N-1}\bigg )\bigg (\frac{1}{h+N-1}\bigg )^{L-1}\left( {\begin{array}{c}N-k-1\\ L-1\end{array}}\right) \bigg (\frac{h+N-2}{h+N-1}\bigg )^{N-k-L}. \end{aligned}$$

Note that the term $\left( \frac{h+N-2}{h+N-1}\right) ^{N-k-L}$ ensures that only *L* hawks are present on $H_1$’s home patch, with the remaining $N-k-L$ hawks elsewhere. $\alpha _{L}$ must be weighed by the number of hawks in the group, resulting in $\alpha _{L}(\frac{1}{L})V$. However, the cost that the average hawk endures must be weighed by $\left( \frac{L-1}{L}\right) C$. This is then summed over all possible group sizes to cover the entire range, $\sum _{L = 1}^{N-k}\alpha _{L}(\frac{1}{L})V$ and $\sum _{L = 1}^{N-k}\alpha _{L}(\frac{L-1}{L})C$. These expressions can be simplified as follows:

For the reward component:

$$\begin{aligned} \sum \limits _{L = 1}^{N-k}\alpha _{L}(\frac{1}{L}) = \frac{h}{N-k}\bigg (1 -\bigg (\frac{h+N-2}{h+N-1}\bigg )^{N-k}\bigg ). \end{aligned}$$

And for the cost component:

$$\begin{aligned} \sum \limits _{L = 1}^{N-k}\alpha _{L}(\frac{L-1}{L}) = \frac{h}{h+N-1}\bigg (1 -\bigg (\frac{h+N-2}{h+N-1}\bigg )^{N-k-1}\bigg ). \end{aligned}$$

By using similar methods for the other scenarios and combining these expressions, we derive the average fitness of a hawk as:

$$\begin{aligned}&{R + \bigg (1 + \frac{k}{N-k} - \frac{(N-1)(h+N-2)^{N-k-1}}{(h+N-1)^{N-k}} - \frac{k(h+N-2)^{N-k}}{(N-k)(h+N-1)^{N-k}}\bigg )V} \\&\quad -\left( \frac{k-N+1}{h+N-1} - \frac{k}{N-k} + \frac{h(N-k-1)+(N-k-1)(N-1)}{(h+N-1)^{2}}\right. \\&\quad \left. + \frac{k(h+N-2)^{N-k}}{(N-k)(h+N-1)^{N-k}} + \frac{(N-1)(h+N-2)^{N-k-1}}{(h+N-1)^{N-k}}\right) C, \end{aligned}$$

which we re-express as

42 $$\begin{aligned} R + \omega (h, N, k) V - \nu (h, N, k) C. \end{aligned}$$

where

$$\begin{aligned} &  \omega (h, N, k) = {\bigg (1 + \frac{k}{N-k} - \frac{(N-1)(h+N-2)^{N-k-1}}{(h+N-1)^{N-k}} - \frac{k(h+N-2)^{N-k}}{(N-k)(h+N-1)^{N-k}}\bigg ),} \\ &  \nu (h, N, k) = \left( \frac{k-N+1}{h+N-1} - \frac{k}{N-k} + \frac{h(N-k-1)+(N-k-1)(N-1)}{(h+N-1)^{2}}\right. \\ &  \quad \left. + \frac{k(h+N-2)^{N-k}}{(N-k)(h+N-1)^{N-k}} + \frac{(N-1)(h+N-2)^{N-k-1}}{(h+N-1)^{N-k}}\right) . \end{aligned}$$

### Weak selection: dove’s fixation probability

The Dove’s fixation probability under BDB dynamics is given by

43 $$\begin{aligned} \rho _{1}^{B} = \frac{1}{1 + \sum \nolimits _{j=1}^{N-1}\prod \nolimits _{k=1}^{j}\frac{R + \omega V - \nu C}{R + \tau V}}. \end{aligned}$$

We carried out weak selection methods on (43). Consider the expression inside the product term of (43).

$$\begin{aligned} \frac{R + \omega V - \nu C}{R + \tau V} = \frac{1+\frac{A}{R}}{1+\frac{B}{R}}, \end{aligned}$$

where

$$\begin{aligned} A = \omega V - \nu C, \text { and } B = \tau V, \end{aligned}$$

$$\begin{aligned} \frac{1+\frac{A}{R}}{1+\frac{B}{R}}&\approxeq 1 + \frac{A-B}{R}. \end{aligned}$$

The term inside the product of (43) now becomes

44 $$\begin{aligned} \prod \limits _{k = 1}^{j}\bigg (\frac{1+\frac{A}{R}}{1+\frac{B}{R}}\bigg ) \nonumber&= \bigg (1+\frac{A(1)-B(1)}{R}\bigg )\bigg (1+\frac{A(2)-B(2)}{R}\bigg )...\bigg (1+\frac{A(j)-B(j)}{R}\bigg ) \nonumber \\&= 1 + \sum \limits _{k=1}^{j}\bigg (\frac{A(k)-B(k)}{R}\bigg ). \end{aligned}$$

So from Eq. (43)

$$\begin{aligned} \sum \limits _{j=1}^{N-1}\prod \limits _{k=1}^{j} \frac{R + \omega V - \nu C}{R + \tau V}&= \sum \limits _{j=1}^{N-1}\bigg ( 1 + \sum \limits _{k=1}^{j}\bigg (\frac{A(k)-B(k)}{R}\bigg )\bigg ) \\&= N - 1 + \frac{1}{R}\sum \limits _{k=1}^{N-1}\bigg (\omega V - \nu C -\tau V\bigg )\bigg (N-k\bigg ), \end{aligned}$$

which simplifies to

45 $$\begin{aligned} N - 1 + \frac{1}{R}\bigg (\sum \limits _{k=1}^{N-1}(\omega V)(N-k) - \sum \limits _{k=1}^{N-1}(\nu C)(N-k) - \sum \limits _{k=1}^{N-1}(\tau V )(N-k)\bigg ). \end{aligned}$$

By substituting the fitnesses from (41) and (42) and simplifying, we have

46 $$\begin{aligned} &  \sum \limits _{k=1}^{N-1}(\omega V)(N-k) = \bigg (N\left( N-1-\frac{x-x^{N}}{1-x}\right) \nonumber \\ &  \quad +\left( \frac{(N-1)x^{N+1}-Nx^{N}+x}{(x-1)^{2}}\right) \left( \frac{h-1}{h+N-2}\right) \bigg )V, \end{aligned}$$

47 $$\begin{aligned} &  \sum \limits _{k=1}^{N-1}(\nu C)(N-k) = \bigg (\left( \frac{1-h}{h+N-2}\right) \left( \frac{(N-1)x^{N+1}-Nx^{N}+x}{(x-1)^2}\right) \nonumber \\ &  \quad - \frac{1}{2}N(N-1) + N\left( \frac{x - x^{N}}{1-x}\right) \bigg )C, \end{aligned}$$

48 $$\begin{aligned} \sum \limits _{k=1}^{N-1} \bigg (\tau V \bigg ) \bigg (N-k\bigg ) = -\bigg (&N(N-1)(h+N-2)^{N-1}(h+N-1)^{-N} \nonumber \\&+ \frac{h-1}{h+N-2}\bigg (\frac{(N-1)x^{N+1}-Nx^{N}+x}{(x-1)^2}\bigg ) \nonumber \\&+ \frac{N(N-1)}{h+N-2}\bigg (Nx^{N}H[N-1, \frac{1}{x^{k}}] - \frac{x-x^{N}}{1-x}\bigg ) \bigg ), \end{aligned}$$

where

49 $$\begin{aligned} H[N-1, a] = \sum \limits _{k=1}^{N-1}\frac{a^{k}}{k} \text { and } x = \frac{h+N-2}{h+N-1}. \end{aligned}$$

By inserting (46), (47) and (48) into (45), we arrive at the following

50 $$\begin{aligned}&N - 1 + \frac{N}{R}\bigg (\bigg ((N-1-\frac{x - x^{N}}{1-x}) + (N-1)\bigg (\frac{(h+N-2)^{N-1}}{(h+N-1)^{N}}\bigg )\nonumber \\&\quad (NH[N-1,1] + 1 - N) \bigg . \nonumber \\&\quad \bigg . - \frac{(N-1)}{h+N-2}\left( Nx^{N}H\left[ N-1,\frac{1}{x^{k}}\right] - \frac{x-x^{N}}{1-x}\right) \bigg )V \nonumber \\&\quad - \bigg (\left( \frac{1-h}{h+N-2}\right) \left( \frac{(N-1)x^{N+1}\!-\!Nx^{N}+x}{N(x-1)^2}\right) \!-\! \frac{1}{2}(N-1) \!+\! \left( \frac{x \!-\! x^{N}}{1-x}\right) \bigg )C\bigg ). \end{aligned}$$

Substituting (50) into (43) and simplifying, we have

51 $$\begin{aligned} \frac{1}{N + \frac{N}{R}\bigg ((\lambda _{1} \!+\! \lambda _{2} \!-\! \lambda _{3} )V - (\lambda _{4})C\bigg )} \approxeq \frac{1}{N}\bigg (1-\frac{1}{R}((\lambda _{1} \!+\! \lambda _{2} \!-\! \lambda _{3})V - (\lambda _{4})C)\bigg ),\nonumber \\ \end{aligned}$$

which is the approximation of the fixation probability of a mutant dove under BDB dynamics where

52 $$\begin{aligned} &  \lambda _{1} = \bigg (N-1-\frac{x-x^N}{1-x}\bigg ), \end{aligned}$$

53 $$\begin{aligned} &  \lambda _{2} = (N-1)\bigg (\frac{(h+N-2)^{N-1}}{(h+N-1)^N}\bigg )(NH[N-1,1]+1-N), \end{aligned}$$

54 $$\begin{aligned} &  \lambda _{3} = \frac{(N-1)}{h+N-2}\bigg (Nx^{N}H[N-1,\frac{1}{x^{k}}]-\frac{x-x^{N}}{1-x}\bigg ), \end{aligned}$$

55 $$\begin{aligned} &  \lambda _{4} = \bigg (\frac{1-h}{h+N-2}\bigg )\bigg (\frac{(N-1)x^{N+1}-Nx^{N}+x}{N(x-1)^2}\bigg ) -\frac{1}{2}(N-1) + \bigg (\frac{x-x^{N}}{1-x}\bigg ).\nonumber \\ \end{aligned}$$

We assumed an infinite well-mixed population i.e. as $N \rightarrow \infty$. We consider each $\lambda _{i}$ for $i \in \{1, 2, 3, 4\}$ and deduce an approximation for each $\lambda _{i}$ as *N* tends to infinity.

For (52), we have,

56 $$\begin{aligned} \lambda _{1} \approxeq \frac{N}{e}. \end{aligned}$$

For (53), we have

57 $$\begin{aligned} \lambda _{2} \approxeq \bigg (\frac{N-1}{e} \bigg )\bigg (\ln (N-1) + \gamma + \frac{1}{N} - 1 \bigg ), \end{aligned}$$

where $\gamma$ is the Euler-Mascheroni constant.

For (54) we have,

58 $$\begin{aligned} \lambda _{3} \approxeq \frac{N}{e}ln(N-1) + \frac{N}{e}f(h) - N + \frac{N}{e}, \end{aligned}$$

where $$f(h) = H[N-1, \bigg (\frac{h+N-1}{h+N-2}\bigg )^{k}] - \ln (N-1).$$

From (55) we have,

59 $$\begin{aligned} \lambda _{4} \approxeq (1-h)(1-\frac{2}{e}) + N(\frac{1}{2}-\frac{1}{e}) + \frac{1}{2}. \end{aligned}$$

By simplifying (56), (57), and (58),

60 $$\begin{aligned} (\lambda _{1} + \lambda _{2} - \lambda _{3} )V = \frac{N}{\textrm{e}}\bigg (\gamma - 1 - f(h, N) \bigg ) + \frac{1}{e}\bigg (2 - \ln (N-1)-\gamma - \frac{1}{N}\bigg ) + N.\nonumber \\ \end{aligned}$$

Substituting (60) and (59) into (51), we have

61 $$\begin{aligned} &  \frac{1}{N}\bigg (1 - \frac{1}{R}\bigg (\bigg (\frac{N}{\textrm{e}}\bigg (\gamma - 1 - f(h, N) \bigg ) + \frac{1}{e}\bigg (2 - \ln (N-1)-\gamma - \frac{1}{N}\bigg ) + N\bigg )V \nonumber \\ &  \quad - \bigg ((1-h)(1-\frac{2}{\textrm{e}})+N(\frac{1}{2}-\frac{1}{\textrm{e}})+\frac{1}{2}\bigg )C\bigg )\bigg ), \end{aligned}$$

which is an approximation of the fixation probability of a mutant dove in an infinite, well-mixed population. The neutrality condition for this case is given by $\rho ^{B}_{1} = \frac{1}{N}$ i.e.

62 $$\begin{aligned} &  \frac{N}{\textrm{e}}\bigg (\gamma - 1 - f(h, N) \bigg ) + \frac{1}{e}\bigg (2 - \ln (N-1)-\gamma - \frac{1}{N}\bigg ) + N\bigg )V\nonumber \\ &  \quad - \bigg ((1-h)(1-\frac{2}{\textrm{e}})+N(\frac{1}{2}-\frac{1}{\textrm{e}})+\frac{1}{2}\bigg )C = 0, \end{aligned}$$

which simplifies to

63 $$\begin{aligned} V = \frac{(\frac{1}{2}-\frac{1}{\textrm{e}})}{(\frac{1}{\textrm{e}}(\gamma - 1 - f(h)) + 1)}C. \end{aligned}$$

By using similar methods to the dove’s fixation probability under BDD dynamics (29), we deduce a similar weak selection approximation given by

64 $$\begin{aligned} \frac{1}{N}\bigg (1-\frac{(N+2w^{*})}{R(N+w^{*})}((\lambda _{1} + \lambda _{2} - \lambda _{3})V - (\lambda _{4})C)\bigg ). \end{aligned}$$

where the neutrality condition remains unchanged.
